# Beyond carrots and sticks. Exploring faculty motivation to join a digital health professions educator program

**DOI:** 10.3389/fmed.2025.1554011

**Published:** 2025-03-05

**Authors:** Marwa Schumann, Martin Lehmann, Harm Peters

**Affiliations:** Dieter Scheffner Center for Medical Education, Dean's Office of Study Affairs, Charité - Universitätsmedizin Berlin, Berlin, Germany

**Keywords:** faculty development, qualitative study, digital health professions education, self-determination theory, intrinsic motivation

## Abstract

**Introduction:**

Faculty development programs in the health professions are essential in addressing the evolving and expanding roles of educators. These programs have become a cornerstone of organizational development and contribute significantly to improving student learning. However, the motivation of faculty to engage in such programs is often challenged by the significant demands of their clinical responsibilities and already busy schedules. This study aims to explore the motivation of teaching health professionals to voluntarily participate in the Digital Health Professions Education (d-HPE) program, a 200-h certification program at the Charité - Universitätsmedizin Berlin to train digital teaching skills and competencies.

**Methods:**

In this qualitative study, we analyzed the motivation letters from faculty members who volunteered to participate in the d-HPE program. We used coding based on Self-Determination Theory (SDT) as a framework for analysis with three main themes: autonomy, competence and relatedness. Within autonomy, the sub-themes were intrinsic motivation and self-directed choices. Competence included the sub-themes of mastery of teaching practices and digital skill development. Relatedness included the sub-themes of interprofessional collaboration and mentorship.

**Results:**

A total of 21 motivational letters were analyzed from two d-HPE cohorts, representing diverse health professional backgrounds and career stages. Analysis of the autonomy theme revealed an intrinsic motivation shaped by early teaching experiences and a self-directed decision to pursue advanced qualifications. The competence theme reflected the need to master evidence-based teaching practices and to develop advanced digital skills, with the COVID-19 pandemic as a driving factor. The relatedness theme demonstrated the importance of inter-professional collaboration and mentorship in promoting educational innovation across disciplines and institutions.

**Discussion:**

The motivation of health professions educators to participate in faculty development programs goes beyond the traditional 'carrots and sticks' of external rewards or punishments constellation; it is rooted in their intrinsic motivation to improve teaching qualifications and fulfill their role in digital education. Despite the demands of a busy clinical and professional work schedule, active engagement in intensive faculty development programs is aligned with the need for interprofessional networking and the evolving demands of digital education.

## 1 Introduction

The rapidly changing landscape of health professions education creates significant challenges for both students and educators, particularly with the increasing integration of digital technologies into education, research, and patient care (1–3). As these changes usually bring both opportunities and challenges, digital education has emerged as a key strategy to equip health professionals with the skills needed to navigate this transformation. Its growing adoption spans all levels of health professions education, from pre- and post-graduate university programs, as well as faculty development, lifelong learning and continuous professional development (2). Faculty participation in development programs to improve digital competencies often depends on intrinsic and extrinsic motivational factors, including the perceived relevance of training to their teaching practice, the opportunity for personal and professional growth, and institutional support (4). However, traditional management approaches commonly rely on the “carrots and sticks model of rewards and punishment that may fail to address the deeper motivational needs, potentially limiting faculty engagement and the long-term impact of such programs. Understanding these motivational factors may be essential for designing effective faculty development programs that promote long-term engagement and successful and sustainable implementation of digital teaching competencies. Therefore, this study seeks to move beyond the “carrots and sticks” framework by exploring intrinsic motivational factors, as outlined by self-determination theory, to better understand what motivates faculty to join and remain engaged in a digital health professions educator program.

Digital education is broad and evolving in nature and is used as an umbrella term for different educational approaches, methods and technologies and is defined as “teaching and learning using digital technologies, ranging from the simple conversion of content into a digital format (e.g., a book into a PDF or HTML format) to the complex use of digital technologies (e.g., mobile education, serious games, virtual patients and virtual reality)” (2). The benefits of digital education generally include flexible and widespread access to content, personalized learning experiences, increased engagement with content and deeper information processing. However, it is also likely to present challenges such as the digital divide (requiring IT infrastructure and digital literacy), higher development and deployment costs, and potential negative emotional effects such as anxiety and feelings of isolation among students and teachers (2).

Following the COVID-19 pandemic, which led to a rapid transition to digital teaching and learning in many educational institutions, there has been an increasing need for faculty development specifically aiming at preparing and supporting health professions educators to deliver effective digital instruction and assessment, maintain student engagement, and foster interprofessional collaboration in a virtual environment (5). Digitally competent faculty are generally better equipped to design and implement educational strategies that meet diverse needs of learners and to navigate the complexities of digital health education (6). As a result, faculty development programs that focus specifically on digital education have received increasing attention for their ability to address the specific learning needs of educators and equip them with skills to teach in a rapidly digitizing environment (4). However, given time pressures associated with clinical practice and service, understanding factors which motivate faculty to voluntarily engage in such faculty development programs is crucial for effective design and implementation in the future (3).

Teaching competencies in health professions education are evolving and expanding to encompass various roles, including the teacher as facilitator and mentor, curriculum developer and implementer, assessor and diagnostician, role model, manager and leader, scholar and researcher, and professional (7, 8). In addition, with recent advances in digital technology, there has been a growing emphasis on digital competence, defined as “the set of knowledge, skills, attitudes, abilities, strategies, and awareness required to use information and communication technology (ICT) and digital media” (9, 10). It includes self-rated competencies (e.g., digital literacy and eHealth literacy), psychological and emotional aspects of using digital technologies (e.g., attitudes and beliefs, confidence and awareness), use of digital technologies (e.g., for general and specific functions) and knowledge of digital technologies (11).

Digital competence is often interchangeably referred to as pedagogical digital competence (PDC), which refers to the ability to consistently apply knowledge, skills, attitudes, approaches to technology and learning theory to plan, deliver, evaluate and continually revise digital education (4). Several digital competency frameworks have been developed for health professions educators to inform the development of faculty training programs in this area (12). However, the digital competencies and support needed by educators vary widely, depending on factors such as pre-existing skills, local conditions and individual needs, which differ from region to region (13).

Despite a widespread need to support these teaching and digital competencies, specialized training programs remain scarce, highlighting a necessity to integrate these skills into faculty development programs to reach health professionals, especially those with heavy workloads and leadership roles (3, 6, 14). Evidence from the literature shows that digital education faculty development interventions have been designed and delivered to a diverse range of healthcare professionals, in a variety of settings, and with a range of different outcome measures (2, 4). The most common approaches used were formal workshops, group work, case studies, discussions and practical exercises or simulations, but relatively little attention was paid to informal and individualized approaches (e.g., peer coaching and collegial support) (8, 15). Evidence from the literature recommends the integration of multi-method strategies in authentic contexts such as experiential learning, role modeling, reflection, and applying evidence to teaching practice to improve digital competencies of teaching health professionals (8, 16, 17). It is also recommended to provide extended longitudinal programs, which could have the advantage of fostering a community of practice, aligning with institutional priorities, promoting educational leadership, and increasing scholarly productivity (8, 18, 19).

To further enhance the effectiveness of faculty development programs, it is essential to consider how learning processes align with key dimensions: cognitive (what to learn), affective or motivational (why to learn), and metacognitive regulation (how to learn) (20). Among these dimensions, motivation plays a critical role in faculty engagement hence focusing on stimulating faculty motivation can have a significant impact on the outcomes of faculty development (FD) programs. Several motivational theories have evolved over time, such as Murray's Need to Achieve Theory (1938), which suggests that motivation is a dynamic construct shaped by time and context rather than a fixed trait (21), Hull's Drive Theory (1943), which suggests that human behavior is driven by needs that must be met to maintain a steady state (22), and Maslow's Hierarchy of Needs (1943), which proposes that human motivation is organized in a hierarchy (23). Other motivational theories include Atkinson's Expectancy-Value Theory (1966), which argues that motivation is influenced by an individual's desire to succeed or avoid failure (24), and Bandura's Social Cognitive Theory (1977), which emphasizes the role of self-efficacy in motivation (25). The Self-Determination Theory (SDT), developed by Edward Deci and Richard Ryan, is one of the current major motivational theories in many fields, including education and health care, and its applications in medical education are gaining increasing interest (26). It was selected for this study because it is particularly well suited to exploring faculty motivation to voluntarily engage in development programs, such as the program that is subject of this study where participation is not externally mandated but driven by personal and professional factors. Given the voluntary nature of the program and the challenges posed by faculty's clinical responsibilities, SDT would allow for a nuanced understanding of internal drivers that influence their decision to participate in an intensive and time-consuming program. This theoretical framework enables the exploration of how intrinsic motivation, rather than external rewards or obligations, plays a key role in faculty engagement, making SDT an appropriate choice for examining the factors influencing participation in faculty development (27, 28).

In Germany, the digitalisation of healthcare has been established by law with the “Digital Healthcare Act” from 2020, and there is a growing need for further training in digital education (13). However, only a few German medical schools have integrated digital health skills into their curricula, often as an elective course that reaches a small number of students (29). There is a growing need to train faculty in digital skills in order to make future courses more accessible to a wider range of students (29). Existing research has predominantly focused on either assessing the digital competencies of health professions educators (11, 30–33) or evaluating the outcomes of faculty development programs designed to enhance digital competencies (15, 34, 35). These evaluations typically assess program effectiveness in terms of teaching effectiveness and successful integration of digital tools into teaching practice. However, there is a gap in the literature regarding the motivations that drive faculty to participate in these programs (36). Addressing this gap seems important for several reasons. First, as motivation directly influences the level of commitment of healthcare educators to their professional development, it is important to design and implement programmes that not only meet institutional goals but also resonate with the personal and professional aspirations of educators. This approach ensures greater commitment and sustained success by investing time and effort in learning new skills, experimenting with innovative teaching methods and overcoming technological challenges (37). A second reason is that faculty development programs require significant institutional investment in terms of resources, time and funding, so it is important for institutions to understand what motivates faculty to participate and remain engaged in these programs (38). Third, motivation research has shown that adult learners, such as educators, have distinct needs to see clear value and relevance to their practice, to have autonomy in their learning, and to feel competent in new skills (39, 40). Finally, sustaining behavioral change in teaching practices, particularly in the context of digital health education, requires more than initial skill development; it requires ongoing motivation to implement and refine new approaches (41, 42). Thus, understanding motivational factors is critical for designing faculty development programs that support the long-term integration of digital competencies into teaching practices.

### 1.1 Aim of the study

The purpose of this study is to explore the motivations of teaching health professional at Charité - Universitätsmedizin Berlin (Charité) to voluntarily enroll in the Digital Health Professions Educator (d-HPE) program, a 200-h interprofessional faculty development program. This research focuses on understanding why educators choose to voluntarily participate in such an intensive program, despite their demanding professional responsibilities.

## 2 Materials and methods

### 2.1 Philosophical assumptions and study design

This study is underpinned by a constructivist epistemology which assumes that knowledge is created through social interaction and that motivation is a complex, contextual phenomenon shaped by personal beliefs, prior experiences and social interactions (43). In line with this paradigm, we adopted an exploratory qualitative research approach to understand the perspectives and motivations of health professions educators applying for the d-HPE program, designed for faculty interested in obtaining a formal qualification in digital education. A qualitative approach also allows for in-depth exploration of nuanced and multifaceted motivational aspects and is increasingly used in the context of motivational theories (44).

### 2.2 Study setting and participants

The study was conducted from May 2023 to July 2024 at the Charité Germany. The sampling frame consisted of educators in the health professions applying for the d-HPE program, a blended learning interprofessional faculty development program delivered over 1 year.

**Figure 1:**
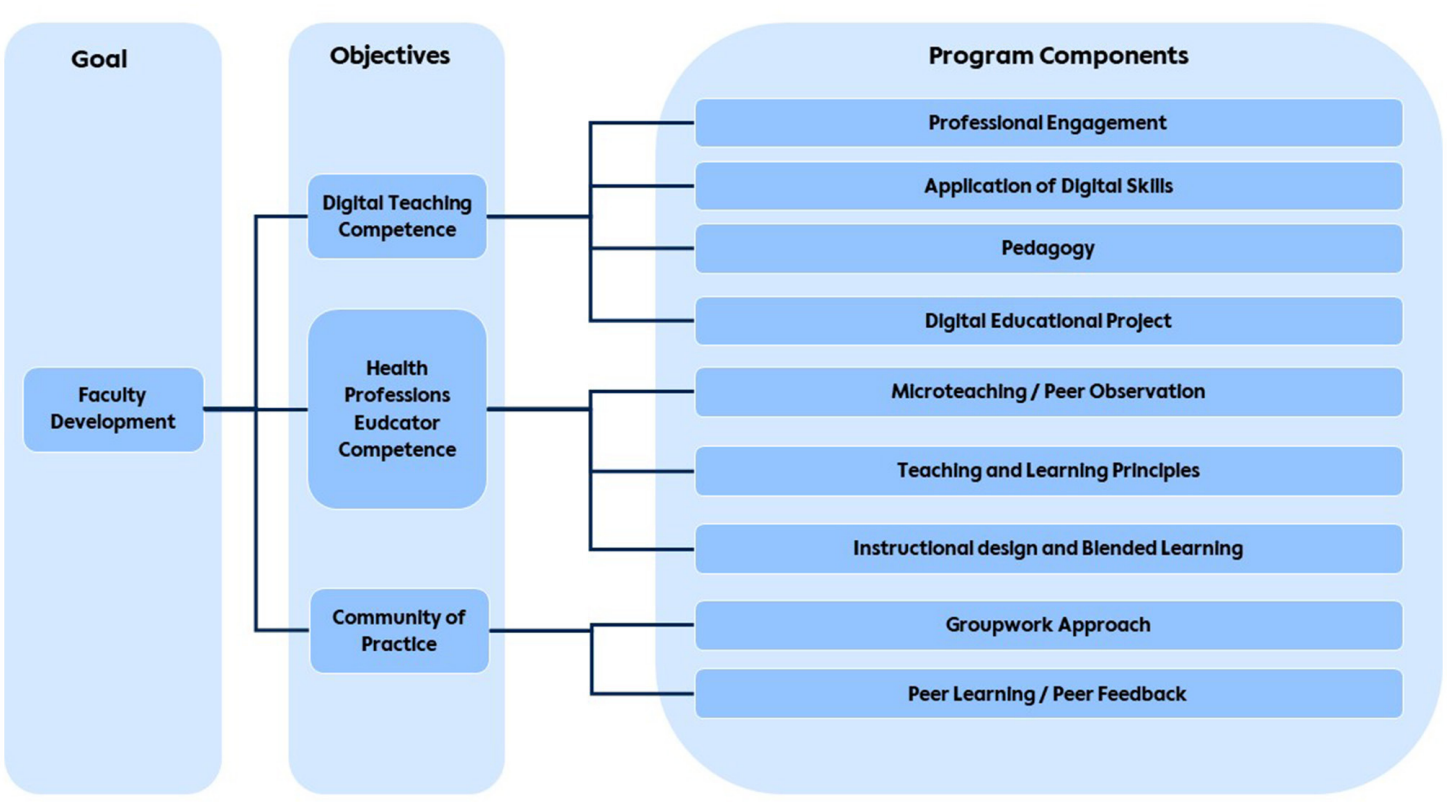
The digital Health Professions Educator Program (d-HPE).

The d-HPE program was developed and implemented as a comprehensive faculty development initiative aimed at equipping healthcare educators from diverse professional backgrounds and career stages at the Charité with digital competencies needed to teach, assess, and mentor in digital education contexts. The program is structured into three progressive modules, comprising a total of 200 teaching units (45 min each), designed to build participants' basic knowledge and skills in addition to advanced application (Figure 1).

Module I (60 teaching units) focuses on the fundamental principles of digital health education, including topics such as constructive alignment, teaching and learning methods, instructional design, and the use of blended learning and online teaching tools. Participants are also introduced to media production techniques where they learn to create digital teaching materials such as screencasts and videos. This module emphasizes reflective practice and peer feedback as essential components of professional development. Module II (60 teaching units) provides more advanced training, enabling participants to engage in the practical application of tools and educational strategies specific to online environments, e.g., the development of virtual patient cases, online seminars and exams, with a particular focus on humanizing online learning environments. Module III (80 teaching units) requires participants to undertake a major digital teaching project, synthesizing and applying their knowledge to the design and implementation of a large-scale digital learning initiative. Throughout this module, participants receive individual mentoring and participate in workshops and journal clubs focused on digital health professions education.

The 12-month program offers a flexible, blended learning approach that includes both online self-study and face-to-face workshops. The final module culminates in the presentation and peer review of participants' digital teaching projects. The program has been co-designed by an interprofessional team of experts in medical education, faculty development, e-learning and interprofessional education at the Charité. A collaborative design process ensured that the program is tailored to the needs of a broader interprofessional range of participants from various areas and health professions. Based on adult learning principles, the program emphasizes collaborative inquiry, where participants learn through shared experiences, and reflective practice, which encourages critical self-assessment and continuous professional development (3).

### 2.3 Data collection

For data collection we used letters of motivation of applicants to the d-HPE program in the two consecutive cohorts of 2023/2024 and 2024/2025. The motivational letters, with an average length of one page, were anonymized and translated into English by the author MS.

### 2.4 Qualitative data analysis

A deductive approach to qualitative data analysis was adopted for this study, which begins with an organizing framework derived from existing literature to provide a structured starting point for analysis, making it both structured and flexible and ensuring that the coding process was grounded in the existing literature (45, 46).

In contrast to conventional qualitative content analysis, used for example in grounded theory, where coding categories are derived directly and inductively from raw data, the current study adopted a directed content analysis approach with the aim of extending the conceptual framework or theory, in our case the Self-Determination Theory (46, 47). Analysis was conducted using Atlas.ti qualitative data analysis (QDA) software (a computerized indexing system, Berlin, Germany) (48). The coding framework was constructed around the three main themes of autonomy, competence, and relatedness (49). The main themes, subthemes, and their definitions are summarized in Table 1.

**Table 1:** Coding framework adapted from Ryan and Deci (49).

**Theme**	**Subthemes**	**The theme explores**
Autonomy	Intrinsic drive	The motivation that arises from personal interest, curiosity, or internal satisfaction in the context of teaching or learning practices.
	Self-directed choices	The motivation derived from autonomy to determine the most appropriate teaching strategies or methods.
Competence	Mastery of teaching practices	The motivation to attain a high level of expertise in the field of teaching.
	Digital Skill development	The motivation to develop or enhance specific teaching skills, with a particular focus on digital and innovative methods.
Relatedness	Interprofessional collaboration	The motivation to collaborate with peers and engage in interprofessional projects.
	Mentorship	The necessity of establishing connections with and helping the forthcoming generation of learners and professionals.

In a collaborative, iterative analysis process, author (MS) carried out the initial analysis based on the first cohort of motivation letters. The analysis was revised by authors (ML) and (HP) and a consensus-building process followed: disagreements in the collaborative analysis process were addressed through a structured and iterative approach. Any differences in analysis were resolved through in-depth discussion to ensure agreement on the coding process. Finally, the analysis of the remaining motivation letters was continued in an iterative process by MS, ML, and HP.

### 2.5 Ethics approval

This study was conducted in compliance with the data protection regulations at the Charité and was approved by the Ethics Committee of the Charité. Anonymity was ensured; a consent form was signed by the participants, who were informed about the study purpose, the use of data collected, and their right to withdraw at any time without consequences for their success in the program.

### 2.6 Reflexivity

The reflexivity was achieved through the maintenance of a research diary, which included notes and comments on the motivation letters as well as interaction between the researchers and the course participants (50). In this case, researchers act as insiders, taking on the role of course development, implementation, and evaluation, as well as being responsible for teaching all participants and providing ongoing support throughout the program. While this role facilitated a deeper understanding of contextual complexities, potential drawbacks emerged, primarily in terms of power dynamics between researchers and participants. Measures were taken to mitigate this, including anonymizing the motivational letters and ensuring that participants' performance was not influenced by their involvement in the study. We also recognize that the researchers' intense involvement in the program may have potentially influenced the data analysis process (51). To address this, we engaged in an iterative process of careful review of the analysis in which initial disagreements were discussed and resolved through collaborative discussions. This approach ensured that consensus was reached and the integrity of the research findings was maintained.

## 3 Results

### 3.1 Participants

A total of 21 motivation letters were analyzed from two d-HPE program cohorts in academic years 2023/2024 and 2024/2025 including anaesthesiology (*n* = 3), pediatrics (*n* = 2), geriatrics (*n* = 2), internal medicine (*n* = 2), emergency medicine (*n* = 2), clinical nursing (*n* = 1), pediatric Surgery (*n* = 1), orthodontics (*n* = 1), radiation oncology (*n* = 1), nephrology (*n* = 1), pharmacology (*n* = 1), IT/scientific computing (*n* = 1), pathology (*n* = 1), medical physics (*n* = 1) and social medicine (*n* = 1).

### 3.2 Autonomy

Regarding the intrinsic drive, the analysis revealed a strong passion for teaching and an enthusiasm for creative and effective teaching methods, e.g., the inherent interest in digital tools combined with a personal passion for drawing and illustrating to enhance medical presentations for research and teaching (Table 2, quote 1). Intrinsic drive is also associated with an early commitment to teaching, either early in the medical career (Table 2, quote 2) or even during the participants' undergraduate years (Table 2, quotes 3 and 4). These examples illustrate how intrinsic motivation, often rooted in personal interests and early experiences, served as a foundational driver for engagement in an intensive faculty development program.

**Table 2:** Autonomy.

	**Quote**	**Participant**
1	“But the digital aspect also appeals to me personally Since the beginning: I already use my passion for drawing and illustrating to prepare medical content for my presentations in research and teaching ”	Male participant, location 7
2	“Since the beginning of my medical career, I have been involved in teaching students with commitment and enthusiasm.”	Female participant, location 10
3	I myself have been involved in teaching since I was a student, including as a tutor. Very early in my medical career, I trained as a medical educator.	Male participant, location 5
4	My passion for teaching began during my studies. I was able to gain my first experience as a lecturer as a member of the anonymous (a student working group).	Female participant, location 1
5	“I would like to optimize my teaching by obtaining a substantial additional qualification”.	Female participant, location 3
6	“I am also convinced that expanding my digital skills will not only enrich my teaching methods, but also advance my personal academic career. The qualification from your program represents an essential investment in my future, enabling me to be a leader in the development and application of innovative teaching strategies”.	Female participant, location 11

In addition to intrinsic motivation, participants made self-directed choices to improve their digital teaching skills and to pursue career advancement and leadership in education. A key motivation was the decision to acquire substantial additional qualifications, reflecting a commitment to continuous improvement in teaching methods (Table 2, quote 5). In addition, the program was seen as an essential investment in personal academic growth, with participants recognizing its potential to enrich their teaching while positioning them to lead the development and implementation of innovative teaching strategies (Table 2, quote 6). This highlights a deliberate and forward-thinking approach, with participants choosing the program to align with their long-term goals of becoming leaders in the evolving field of medical education.

In summary, the analysis of the autonomy theme reveals that faculty members' motivation to engage in the d-HPE program stems from both an intrinsic drive, rooted in a passion for teaching and early teaching experiences, and a self-directed decision to strategically pursue advanced qualifications to improve teaching practice and position themselves as leaders in educational innovation.

### 3.3 Competence

In terms of mastering teaching practice, participants expressed a need to expand expertise and refine evidence-based teaching methods. For some, this need stemmed from an early transition from learner to teacher, which highlighted a need to better understand teaching practices and pursue structured educational training in didactics in order to gain confidence and competence (Table 3, quote 1). Participants also emphasized the importance of continuously improving their pedagogical and instructional skills in order to increase their teaching effectiveness and adaptability (Table 3, quote 2). In addition, grounding teaching on sound evidence while remaining open to experimenting with innovative approaches was highlighted as a key strategy, reflecting a commitment to combining proven pedagogical methods with flexibility and creativity in practice (Table 3, quotes 3 and 4).

**Table 3:** Competence.

	**Quote**	**Participant**
1	“In 2016, after seven years of studying medicine, I went from being a learner to a teacher in one fell swoop. This change of perspective made me realize that I really enjoy teaching, but also that I know relatively little about my new role. And although my growing experience in the many courses that followed gave me a lot of confidence, I was always keen to immerse myself in didactics in a structured way.”	Male participant, location 7
2	“By strengthening and expanding my own didactic and methodological skills, I can further develop my 'own' teaching and lecturing activities.”	Female participant, location 9
3	“I want to base my teaching on sound evidence and to learn and try new approaches.”	Male participant, location 14
4	“My main goal is to use the program to refine my teaching methods so that they not only meet current academic standards, but also increase the interactivity and accessibility of my teaching.”	Female participant, location 11
5	“The restrictions imposed by the Corona pandemic over the last 3 years have impressively demonstrated the importance of innovative and digital approaches to teaching in (higher) education institutions. Acquiring the skills to create digital learning content, such as videos or live online surveys, would be a great asset, as this will certainly play an increasingly important role in knowledge transfer at universities in the future.”	Female participant, location 6
6	“I hope that my participation in the Digital Health program will give me the opportunity to develop and implement my own media-based teaching concepts.”	Male participant, location 13
7	“The goal of the participation is to develop an interactive online course on *anonymized* topics for *anonymous*, which will provide basic knowledge through examples and illustrations and provide feedback to the students.”	Male participant, location 15

Regarding the development of digital skills, participants highlighted the challenges and opportunities presented by the COVID-19 pandemic, emphasizing the need to adopt innovative digital approaches to enhance knowledge transfer, such as creating digital learning content like videos and live online surveys, as essential tools for future educational success (Table 3, quote 5). In addition, there was a motivation to use the dHPE program to help develop and implement personalized media-based teaching concepts or interactive online courses tailored to specialized topics to support student engagement and provide real-time feedback (Table 3, quotes 6 and 7).

In summary, the theme of competence underscores participants' motivation to master teaching practice in general and to develop advanced digital skills in particular, acknowledging the role of the COVID pandemic in accelerating an adoption of innovative teaching strategies and reflecting a dual focus on evidence-based methods and innovative approaches.

### 3.4 Relatedness

Regarding interprofessional collaboration, motivation to engage in collaborative learning and interdisciplinary programs was particularly evident, with some participants emphasizing the collaborative nature of their fields, making interprofessional faculty development programs and collaborative learning opportunities essential to advancing interprofessional collaboration in therapy, research, and teaching (Table 4, quote 1). Others expressed a commitment to developing teaching methods and tools tailored to the needs of interprofessional health care teams, e.g., creating and implementing digital teaching and learning tools for interprofessional contexts as a key goal for applying to the program (Table 4, quote 2). Similarly, participants recognized the role of interprofessional networking in improving educational practice and promoting innovative, future-oriented teaching methods in an interprofessional skills lab (Table 4, quote 3). Other participants highlighted the potential of such collaboration to initiate interprofessional teaching projects and drive development of digital teaching approaches, emphasizing the critical role of interprofessional exchange (Table 4, quote 4).

**Table 4:** Relatedness.

	**Quote**	**Participant**
1	“*Anonymous* is a particularly interprofessional field; we communicate, treat and research with other areas of human medicine, medical technology and materials development. This makes an interprofessional program and joint learning particularly interesting for us in order to establish further cooperation in therapy, research and teaching.”	Male participant, location 4
2	Another key objective is to develop and implement digital teaching and learning tools specifically tailored to the needs of interprofessional healthcare professionals.	Female participant, location 11
3	“My goal is to further develop innovative, future-oriented learning and teaching methods in the *anonymous* at the Charité. I am convinced that the exchange and networking of faculty and teaching staff through programs such as this can significantly advance teaching, and I would like to be a part of it.”	Female participant, location 1
4	“My goals for participating in the program are networking and exchanging experiences with other teachers at the Charité to initiate interprofessional teaching projects and to work together on the further development of digital teaching concepts.”	Male participant, location 12
5	“I would like to provide the next generation of health professionals with adequate training and digital and online learning materials.”	Female participant, location 3
6	“Of course, I would also like to pass on the knowledge I have gained to interested colleagues, not only in *anonymous*, and thus support the integration of the various possibilities of digitalization into university didactics.”	Female participant, location 6
7	“In addition, I would like to pass on the knowledge gained in such a project not only to interested colleagues already working in the field of anonymous, but also to support other digital didactic projects at universities.”	Male participant, location 6

Mentorship was highlighted as a central component of motivation, with participants aiming not only to support the development of students, but also to actively contribute to professional growth of their professional peers, ensuring that advances in education and digital instructional methods are widely shared and implemented. Passing on knowledge and skills to future generations of health professionals reflects a commitment to supporting peers and junior colleagues with digital tools in a rapidly evolving educational landscape (Table 4, quote 5). Application to the d-HPE program was also motivated by an intention to integrate digitization into university teaching by disseminating any knowledge gained to colleagues, further underscoring the cascading effect of mentorship in fostering innovation across disciplines (Table 4, quote 6). Other participants noted the value of sharing insights with colleagues in specialized fields while also contributing to broader digital teaching projects at universities (Table 4, quote 7).

In summary, the theme relatedness emphasizes the importance of interprofessional collaboration and mentorship to promote educational innovation, professional development and the dissemination of advanced teaching practices across disciplines and institutions.

## 4 Discussion

The motivation of teaching health professionals to deliberately participate in an extensive faculty development program, despite the significant demands of their clinical roles, is a critical factor to the success and sustainability of such initiatives. This study explored motivations of health professions faculty to engage in a voluntary faculty development program, the d-HPE at the Charité Berlin. The theoretical framework of self-determination theory, with its three dimensions of autonomy, competence and relatedness, provided a deeper understanding of the personal and professional factors that drive participation in such a faculty development program. In the following sections, we will discuss our findings in the context of the literature, highlighting the importance of intrinsic motivation and the need for teachers to feel supported in their professional development. In addition, we will derive implications on how to motivate faculty to engage in similar faculty development programs based on the findings identified in our sample of motivation letters.

Overall, the results indicate that intrinsic motivation plays an important role in attracting faculty to participate in faculty development initiatives, moving beyond the “carrots and sticks” of extrinsic motivations commonly highlighted in the literature, such as scholarships, awards, promotions, incentives, or coercion by department heads (36). While intrinsically motivated faculty may be more likely to use best teaching practices, extrinsically motivated faculty may choose less effective strategies as their goal is the shortest path to outcome completion (28). Autonomy and self-direction were associated with increased motivation, so rather than focusing solely on institutional and functional needs, addressing faculty members' perceived needs for autonomy in planning their own professional development path led to higher levels of curiosity to learn and try new things, resulting in increased sustainability and success of faculty development initiatives (52–55). Conversely, the limited autonomy experienced by some educators appeared to inhibit the long-lasting impact of continuing professional development projects, despite the voluntary nature of attendance (56, 57). Autonomous motivation was also found to predict greater incorporation of effective teaching strategies and instructional clarity, as well as collaborative learning, by healthcare professions educators (28). It is therefore recommended that teachers' engagement in professional development activities should be driven by their own determination, alignment with their personal aspirations or values, confidence in their ability to acquire new skills, and autonomy in shaping their own professional development trajectories, rather than by extrinsic sources of motivation.

Early teaching experiences serve as important sources of motivation for engaging in faculty development, which is consistent with the literature suggesting that early teaching experiences can play a critical role in shaping educators' long term commitment to teaching (58, 59). This is particularly important in shaping the identity as a health professions educator, as prior to teaching, students do not make an explicit connection between teaching and being a physician (or maybe better) “health professional” (58). Thus, our findings underscore an importance of promoting early teaching experiences as it can significantly increase motivation to participate in faculty development programs and promote an identity as a teacher (54).

Regarding the competence theme, our results show that motivation to master evidence-based teaching practices and develop advanced digital skills encourages health professionals to engage in faculty development activities, which is consistent with previous research (36). In this context, digital competence, characterized by a desire to learn and develop professionally, is crucial for institutions to develop digital competence policies and initiatives, plan professional development and integrate technology into teaching practice (60, 61). However, research has shown that motivation to learn and teach digital competence is not always directly related to training received; it is also influenced by other factors such as “working climate” and institutional support (62). A key implication, therefore, is that faculty development and competence are mutually reinforcing. The participation in faculty development activities enhances teachers' digital competence, while the desire to improve one's own competence serves as a key motivator for engaging in such initiatives (61).

Regarding the relatedness theme, participants showed a strong appreciation of interprofessional collaboration, recognizing its value in networking, developing collaborative skills and fostering a better understanding of different professional roles. These findings are consistent with recent literature advocating a growing need for interprofessional faculty development programs and describing relatedness as the “enjoyment” of working with and learning from others (63, 64). This reinforces the notion that faculty development should foster a sense of community and support, and that digital interprofessional education is needed across the continuum of undergraduate, postgraduate and faculty development (65, 66). In addition, the integration of digital tools into interprofessional teaching contexts addresses the frequent lack of advanced digital skills among health professional educators, particularly in collaborative and interdisciplinary settings (67). Participants in our study emphasized the importance of creating and implementing digital teaching tools tailored to interprofessional health care teams, reflecting a commitment to bridging this digital skills gap.

Mentorship emerged as another key theme, with participants in our study expressing a strong desire to support the professional development of colleagues and students through mentoring initiatives. This focus on mentorship reflects a commitment to long-term professional development, with teachers acting as catalysts for innovation by passing on knowledge and skills to both students and colleagues. Just as formal faculty development programs—such as workshops—provide structured opportunities for professional growth, mentoring serves as an important, often informal, approach to fostering long-term development (68). This highlights the critical role of mentorship in promoting sustainable professional development and reinforces the idea that faculty development is not only about individual growth and promotion, but also about creating communities of practice and cultivating a culture of continuous learning and excellence in healthcare education.

In summary, faculty are intrinsically motivated to engage in intensive programs in addition to their clinically demanding lifestyles and daily responsibilities when the faculty development initiatives combine formal and informal learning elements to provide a flexible, longitudinal learning experience in an interprofessional setting. The motivation for faculty to participate in such intensive programs goes beyond carrots and sticks, i.e., external rewards and punishments, and is driven by their personal commitment to professional development, the desire to acquire new skills, and the opportunity to collaborate with colleagues across disciplines, all of which contribute to their long-term satisfaction and development as educators. The carrots and sticks metaphor is consistent with the findings of the current study, where faculty participation is driven by internal factors (autonomy, competence and relatedness) rather than external rewards or punishments. It is also consistent with the theoretical framework of Self-Determination Theory (SDT) used in the study, which emphasizes intrinsic motivation over external rewards and punishments. The use of the metaphor also aims to effectively challenge traditional management approaches that rely heavily on rewards and punishments and reflects the modern understanding of professional motivation in education. The integration of structured workshops with peer coaching and collaborative networking to promote personal and professional growth helps to increase motivation. By fostering interprofessional collaboration and sustained engagement, the faculty development program not only improves individual teaching practice, but also supports the development of a culture of continuous learning, ultimately bridging the digital skills gap and advancing healthcare education.

While this study provides valuable insights into motivations of health professions educators to engage in a digital health professions education program, some limitations need to be acknowledged. First, the study relies on a small sample size of 21 motivational letters from two d-HPE cohorts, which may limit the generalisability of the findings to a broader population of health professions educators. In addition, all participants were from a single institution, Charité Berlin, where the 21 teachers are known within the institution for their participation in the programme. To protect their confidentiality and adhere to ethical research standards, we anonymised detailed demographic data such as place of work and specialty. However, this limits the level of contextual detail in the findings and their wider applicability to different educational settings with different institutional cultures and resources. In addition, the analysis is based on self-reported data, which introduces the potential for social desirability bias. Participants may have framed their motivations in ways that they perceived to be more socially acceptable or in line with the goals of the programme. While collaborative discussions during the analysis helped to ensure rigor and consensus, the inclusion of additional methods, such as interviews or focus groups, in future research could provide richer and more triangulated data. The lack of a comparison group of faculty who did not participate in the d-HPE program is another limitation, as this would have allowed for a deeper exploration of the unique factors driving participation relative to broader faculty development needs. Future research should aim to address these limitations by including a larger and more diverse sample from multiple institutions, incorporating a mixed methods approach, and including a comparison group to provide a more comprehensive and generalizable understanding of faculty motivations in similar programs.

## 5 Conclusions

Motivation of teaching health profession faculty to participate voluntarily in an intensive faculty development program is primarily driven by intrinsic motivational factors, particularly the desire for professional development and mastery of digital teaching skills. Rather than being swayed by the traditional “carrots and sticks” of external rewards or pressures, faculty are motivated by the opportunity to improve their teaching practice and contribute to educational innovation in digital health, despite the challenges posed by their busy clinical workloads. The findings also highlight the importance of interprofessional collaboration and mentorship in fostering a sense of belonging and supporting continuous professional development. Effective faculty development programs should prioritize autonomy, competence and relatedness to enhance engagement and align with the evolving demands of digital education. In addition, there is a need for faculty development initiatives that not only respond to external pressures, but also cultivate intrinsic motivations to ensure sustained faculty engagement and to facilitate professional growth.

## Data Availability

The raw data supporting the conclusions of this article will be made available by the authors, without undue reservation.
